# Assessment of body mass index in a pediatric population aged 7–17 from Ukraine according to various international criteria–A cross-sectional study

**DOI:** 10.1371/journal.pone.0244300

**Published:** 2020-12-18

**Authors:** Katarzyna Dereń, Justyna Wyszyńska, Serhiy Nyankovskyy, Olena Nyankovska, Marta Yatsula, Edyta Łuszczki, Marek Sobolewski, Artur Mazur

**Affiliations:** 1 Institute of Health Sciences, Medical College of Rzeszow University, Rzeszow, Poland; 2 Pediatrics Department #1, Danylo Halytsky L’viv National Medical University, L’viv, Ukraine; 3 Department of Pediatrics and Neonatology, Danylo Halytsky L’viv National Medical University, L’viv, Ukraine; 4 Faculty of Management, Rzeszow University of Technology, Rzeszow, Poland; 5 Institute of Medical Sciences, Medical College of Rzeszow University, Rzeszow, Poland; San Raffaele Roma Open University, ITALY

## Abstract

Childhood obesity is one of the most serious public health problems. The prevalence of obesity among children is increasing and may negatively affect their immediate health, but it can also lead to obesity in adulthood. The aim of the study was to compare BMI cut-off points by examining three main international references: the World Health Organization (WHO), the International Task Force Obesity (IOTF) and the US Center for Disease Control and Prevention (CDC). Ultimately, the study group consisted of 18,144 children and adolescents aged 6.5–17.5 years. Body mass was measured on medical scales with an accuracy of ± 100 g and height measurement was taken using a height meter with an accuracy of ± 0.1 cm three times. Underweight, overweight and obesity were calculated according to WHO, IOTF and CDC BMI international references. There were differences in the incidence of underweight between the classifications: 16.8% according to IATF, 5.3% according to WHO and 9.9% according to CDC. There were also differences in the incidence of overweight and obesity between the classifications: 13% according to IOTF, 19.7% according to WHO and 14.1% according to CDC. In the CDC and WHO studies, a significantly higher prevalence of childhood obesity (4.0% and 4.7%, respectively) was observed compared with IOTF (2.1%). The prevalence of overweight and obesity in this study was higher among boys compared to girls. However, estimates of prevalence of overweight and obesity differ in methods and reference cut-off points. Higher prevalence was obtained in IOTF classification, followed by the WHO and CDC classification.

## Introduction

Overweight and obesity can be defined as abnormal or excessive fat accumulation that can be harmful to health [1]. Childhood obesity is one of the major public health problems. The prevalence of obesity among children is increasing and may negatively affect their immediate health, but it can also lead to adult obesity, which is often conducive to chronic diseases including diabetes, hypertension, hyperlipidemia and cardiovascular diseases, even at a young age [2,3].

Obesity is increasing worldwide, affecting children and adults in both developed and developing countries [4,5]. Thirty-nine percent of adults at the age of 18 years and older were overweight in 2016 and 13% were obese. Thirty-eight million children under the age of 5 years were overweight or obese in 2019 [6]. Over 340 million children and adolescents aged 5–19 were overweight or obese in 2016. Most of the world's population lives in countries where overweight and obesity kill more people than underweight [6].

Body mass index (BMI) is a simple index of the relationship between body mass and height, which is often used to identify overweight and obesity. It is widely used to classify overweight and obesity in adults [3]. In 2006, the World Health Organization (WHO) published a growth standard for children up to five years of age based on data from healthy children around the world [7]. Overweight and obesity for children aged 5–19 are defined as follows: overweight is defined as weight-for-height greater than 1 standard deviation above the WHO growth reference standard median and obesity is defined as weight-for-height greater than 2 standard deviations above the WHO growth reference standard median [6]. In 2000, the International Obesity Task Force (IOTF) used data from six large representative cross-sectional height studies (Brazil, UK, Hong Kong, the Netherlands, Singapore and the United States) to develop an international definition of child overweight and obesity [8]. In 2002, updated cut off points and growth charts for the United States were published by Centers for Disease Control and Prevention (CDC) [9].

Ukraine is characterized by an increasing prevalence of overweight and obesity in children, and recent evidence also shows a significant prevalence of underweight. Underweight is often viewed as a sign of malnutrition and poverty in developing countries and in developed countries, it may be associated with eating disorders. In Ukraine, the incidence of underweight according to WHO was 5.3% [10]. Underweight and overweight in children are associated with distinct adverse consequences in childhood, and both are understood to contribute to the development of cardiometabolic diseases in adulthood [11]. Given these far-reaching public health implications, it is imperative to identify and address both underweight and overweight in children. No research to date has systematically compared both the classification of underweight and overweight in Ukraine using the three international sources examined here.

This paper compares the cut-off points for BMI, examining three main international references: the World Health Organization (WHO), the International Task Force on Obesity (IOTF) and the US Center for Disease Control and Prevention (CDC). This study will provide evidence about differences between common international references when classifying child and adolescent weight status. The aim was to analyze the prevalence of underweight and obesity in children and adolescents from Ukraine and observing the degree of concordance to which different classifications allow comparison between different surveys and countries. This will provide updated data on BMI, and prevalence of undrweight, overweight and obesity in children and adolescents in Ukraine.

## Materials and methods

### Participants

The study was conducted in randomly selected primary, secondary and high schools of Ukraine. Sample sizes were determined using EPI INFO (StatCalc, Atlanta, Georgia, US) software. The research was carried out in randomly selected schools in Ukraine between 2018–2019. Approximately 30,000 children and adolescents were selected from 50 primary, secondary and high schools from 20 districts of Ukraine. All students from the selected schools were invited to participate in the study, and 19,745 parental approvals were received for participation of their children in the study. Inclusion criteria were: obtaining informed consent from parents or guardians, enrolling in the selected schools, a functional state that allow for self-maintenance of a standing position, not taking medication affecting body mass, and an age between 7 and 17 years old. Out of 19,745 students 1601 students were excluded from the study for the following reasons: failure to meet inclusion requirements (n = 664) and absence from school on assessment days (n = 937). Ultimately, the study group consisted of 18,144 children and adolescents aged 6.5–17.5 years. Among the representative sample of 18,144 students who were included in this study, 8717 (48%) were boys and 9427 (52%) were girls.

The child's exact age was calculated from the difference between the date of the examination and the date of birth. Exact ages were classified into age groups x (where x = 7 to 17) by placement of exact age within the interval (x—0.5 years, x + 0.5 years).

### Anthropometric measurements

The examinations were carried out in the offices of school nurses in the morning. Assessments were performed by the same group of researchers that have extensive experience. Height and weight were measured for each participant. These measurements were made with the students in their underwear and without shoes. Each measurement was taken as the mean of three consecutive measurements. In children, measurements of body mass and height were performed in triplicate. Body mass was measured with the medical scale RADWAG WPT 60/150 (RADWAG, Radom, Poland) with an accuracy of ± 100 g. Height of the children was measured with a stadiometer attached to the scales with an accuracy of ± 0.1 cm. All scales were tared using a standardized 1 kg weight.

Mean values of height and weight were obtained from three measurements in order to calculate body mass index (BMI) according to the formula: BMI = body mass in kg/height in m^*2*^. Three criteria were used in our study to define childhood underweight, overweight and obesity. The International Obesity Task Force (IOTF) reference [8], provided sex- and age-specific BMI cut-off points that correspond to the BMI cut-off points used in defining underweight, overweight and obesity in adults, 18.5, 25 and 30, respectively. The World Health Organization (WHO) child growth standard, based on the WHO Reference 2007 [12], children aged 6–19 years are overweight and obese with excess weight over 1 SD and 2 SD, respectively, and underweight under 2 SD. The US Center for Disease Control and Prevention (CDC) 2000 cut off points were used to define underweight, overweight and obesity: 5th, 85th and 95th BMI percentile, respectively [13].

### Statistical analysis

The number and percentage of people with the BMI classification defined according to individual standards (WHO, CDC, IOTF) was determined; this summary was prepared by age and gender of children. In order to compare the prevalence of individual BMI categories among girls and boys, a chi-square test of independence was used and prevalence ratios were calculated with a 95% confidence interval.

The z-scores were constructed based on the Box-Cox transformation (using WHO formulas) in such a way that a value of 0 corresponds to the median of the standard BMI distribution for a given age and sex. The z-score values for girls and boys were compared using the t-test for independent samples.

Statistical analysis was performed using the STATISTICA 13.0. Statistical significant differences are denoted by *, ** or *** (for *p* ≤ 0.05; *p* ≤ 0.01 and *p* ≤ 0.001 respectively).

### Ethics

Written informed assent was obtained from parents before participation in the study. The study was approved by the Bioethics Committee of the Medical Department of the University of Rzeszów, decision no 2015/12/15 on 2 December 2015, and it was conducted in accordance with ethical standards stated in the Declaration of Helsinki.

## Results

Table 1 presents the values of descriptive statistics of the distribution of somatic features and BMI index in relation to sex and age of the examined children, as well as the number of examined children broken down by age and sex. The most numerous group was represented by boys and girls aged 8 (13.4% and 12.7%, respectively). There was also a large proportion of boys and girls aged 9 and 10. The least numerous groups among both girls and boys were 16 and 17-years-olds, 5% and 2.8% respectively for girls and 3.9% and 1.7% respectively for boys.

**Table 1:** Distribution of height, weight and BMI in age groups.

**Age [years]**	*N*		Height [cm]			Weight [kg]			BMI [kg/m^2^]		
		%	$\bar{x}$	*s*	95% c.i.	$\bar{x}$	*s*	95% c.i.	$\bar{x}$	*s*	95% c.i.
**boys**											
**7**	989	11.3	126.0	6.5	125.6–126.4	25.3	4.7	25.0–25.6	15.9	2.4	15.8–16.1
**8**	1164	13.4	131.0	6.9	130.6–131.4	28.3	5.7	28.0–28.7	16.4	2.5	16.3–16.6
**9**	1013	11.6	136.7	7.7	136.2–137.1	31.7	6.4	31.3–32.1	16.9	2.6	16.8–17.1
**10**	1039	11.9	141.8	8.0	141.3–142.3	35.8	7.8	35.3–36.3	17.7	3.0	17.5–17.9
**11**	953	10.9	146.8	7.9	146.3–147.3	38.7	8.0	38.2–39.2	17.9	2.9	17.7–18.1
**12**	874	10.0	153.0	8.7	152.4–153.6	43.4	9.3	42.8–44.1	18.5	3.1	18.2–18.7
**13**	864	9.9	159.4	8.8	158.9–160.0	48.3	10.1	47.6–49.0	18.9	2.9	18.7–19.1
**14**	706	8.1	166.0	9.4	165.4–166.7	54.2	11.1	53.4–55.1	19.5	2.9	19.3–19.8
**15**	629	7.2	171.4	8.8	170.7–172.1	59.6	11.1	58.7–60.4	20.2	2.9	20.0–20.4
**16**	341	3.9	176.5	7.9	175.7–177.4	64.3	10.2	63.3–65.4	20.6	2.5	20.3–20.8
**17**	145	1.7	177.6	7.9	176.4–178.9	67.8	11.4	66.0–69.7	21.4	2.8	21.0–21.9
**Total**	8717	100									
**girls**											
**7**	1004	10.7	125.1	6.6	124.7–125.5	24.6	4.6	24.3–24.9	15.7	2.3	15.5–15.8
**8**	1196	12.7	130.4	6.9	130.0–130.8	27.3	5.0	27.0–27.5	16.0	2.4	15.8–16.1
**9**	1111	11.8	136.2	7.4	135.7–136.6	30.5	6.1	30.2–30.9	16.4	2.5	16.2–16.5
**10**	1072	11.4	141.5	7.4	141.1–141.9	33.7	6.7	33.3–34.1	16.8	2.7	16.6–17.0
**11**	1009	10.7	147.5	8.1	147.0–148.0	37.4	7.1	36.9–37.8	17.1	2.6	17.0–17.3
**12**	948	10.1	153.3	8.2	152.7–153.8	41.6	7.8	41.1–42.1	17.6	2.6	17.5–17.8
**13**	911	9.7	159.1	6.8	158.6–159.5	46.6	8.1	46.1–47.1	18.4	2.6	18.2–18.5
**14**	792	8.4	162.6	6.5	162.2–163.1	50.2	7.3	49.7–50.7	19.0	2.5	18.8–19.2
**15**	648	6.9	164.4	6.3	163.9–164.9	52.3	7.5	51.7–52.9	19.3	2.4	19.1–19.5
**16**	476	5.0	165.4	5.9	164.9–165.9	53.3	6.7	52.7–53.9	19.5	2.2	19.3–19.7
**17**	260	2.8	165.6	6.1	164.8–166.3	54.1	7.5	53.2–55.0	19.7	2.4	19.4–20.0
**Total**	9427	100									

$\bar{x}$—mean, *s*–standard deviation, 95% c.i.–confidence interval.

Children's BMI was classified according to three standards—WHO, CDC and IOTF. All classifications were made taking into account sex and age (in months) of the children (Table 2). The classification of children according to BMI for different reference values gives different results. The percentage of obese children ranges from 2.1% for the IOTF classification, 4.0% for CDC and 4.7% for WHO. There were large differences in assessing underweight. The WHO classification showed that the number in both girls and boys with body mass deficiency was similar (5.4% and 5.2%, respectively). According to the WHO definition, the incidence of obesity among boys was 7.0%, and 2.6% in girls. Ten -year-olds (11.1%) were the most obese among boys, and 7-year-olds (5.3%) among girls. The prevalence of overweight based on the WHO definition was significantly higher in boys (19.0%) than in girls (11.3%). The highest percentage of overweight boys was among 9-12-year-olds, and the most overweight girls were 8-9-year-olds. It was observed that with age the percentage of children with normal body mass increased, regardless of sex and classification.

**Table 2:** Prevalence (%) of underweight, overweight or obesity among Ukrainian children and adolescents based on three international BMI references.

Age group (years)	Underweight %			Normal %			Overweight %			Obesity %		
	IOTF	WHO	CDC	IOTF	WHO	CDC	IOTF	WHO	CDC	IOTF	WHO	CDC
**All**												
**7**	18.9	6.2	14.7	66.0	71.7	67.3	11.0	15.2	11.3	4.1	6.9	6.7
**8**	18.0	5.4	11.9	65.5	69.5	69.1	13.3	17.9	12.8	3.2	7.2	6.1
**9**	16.9	5.7	10.7	66.8	68.9	71.0	14.0	19.0	13.7	2.3	6.4	4.6
**10**	15.0	5.3	8.5	69.0	69.6	74.0	12.6	17.8	11.7	3.4	7.3	5.8
**11**	16.5	5.7	10.1	70.7	72.7	76.2	11.4	17.0	10.2	1.4	4.6	3.5
**12**	17.6	6.6	10.6	70.5	75.1	76.9	11.0	14.4	9.4	0.9	3.9	3.1
**13**	15.8	5.0	7.9	73.5	78.3	81.4	9.6	14.4	8.7	1.1	2.4	2.0
**14**	15.3	4.1	6.9	75.5	83.4	84.0	8.2	10.5	7.2	1.0	2.0	1.9
**15**	15.4	3.8	6.2	76.6	84.7	86.1	7.2	10.0	6.1	0.8	1.5	1.6
**16**	17.7	3.5	7.6	76.4	89.7	87.3	5.4	6.1	4.3	0.5	0.6	0.9
**17**	20.0	2.7	8.4	73.6	89.1	86.7	5.7	7.2	4.0	0.7	1.0	1.0
**Total**	**16.8**	**5.3**	**9.9**	**70.2**	**75.0**	**76.1**	**10.9**	**15.0**	**10.1**	**2.1**	**4.7**	**4.0**
**Boys**												
**7**	17.4	7.3	14.0	66.8	67.2	65.1	11.8	16.9	13.1	3.9	8.6	7.8
**8**	16.6	5.2	10.6	65.5	65.5	66.5	13.8	19.7	14.7	4.1	9.6	8.2
**9**	14.4	6.7	10.0	66.8	62.8	67.0	15.9	21.4	16.9	2.9	9.1	6.1
**10**	11.6	4.8	7.4	67.9	61.6	68.3	16.2	22.5	15.9	4.2	11.1	8.4
**11**	13.3	4.8	8.4	70.7	66.5	72.5	14.1	21.2	13.6	1.9	7.5	5.5
**12**	13.6	5.4	9.2	69.5	68.2	72.3	15.4	20.0	13.5	1.5	6.4	5.0
**13**	11.3	4.6	7.6	74.9	72.5	78.1	12.2	19.1	11.1	1.6	3.8	3.1
**14**	10.6	4.5	6.5	75.4	76.2	79.9	12.5	15.6	10.1	1.6	3.7	3.5
**15**	10.3	3.3	6.0	78.2	78.9	82.8	10.0	15.1	8.1	1.4	2.7	3.0
**16**	10.6	2.3	6.5	78.3	85.3	83.9	10.3	11.4	8.2	0.9	0.9	1.5
**17**	9.0	3.4	6.2	78.6	81.4	82.8	11.7	13.8	9.7	0.7	1.4	1.4
**Total**	**13.4**	**5.2**	**8.9**	**70.4**	**68.9**	**72.2**	**13.6**	**19.0**	**13.1**	**2.6**	**7.0**	**5.7**
**Girls**												
**7**	20.4	5.1	15.4	65.1	76.2	69.5	10.2	13.5	9.5	4.3	5.3	5.6
**8**	19.4	5.6	13.3	65.5	73.5	71.6	12.8	16.1	11.0	2.3	4.8	4.1
**9**	19.3	4.9	11.4	66.8	74.4	74.5	12.2	16.8	10.9	1.7	3.9	3.2
**10**	18.3	5.8	9.6	70.0	77.3	79.5	9.2	13.2	7.6	2.5	3.7	3.4
**11**	19.5	6.5	11.7	70.7	78.6	79.7	8.8	13.0	6.9	1.0	1.9	1.7
**12**	21.2	7.7	11.9	71.5	81.5	81.2	6.9	9.2	5.6	0.4	1.6	1.3
**13**	20.1	5.4	8.2	72.2	83.8	84.5	7.1	9.9	6.4	0.5	1.0	0.9
**14**	19.4	3.7	7.2	75.6	89.9	87.6	4.4	5.9	4.7	0.5	0.5	0.5
**15**	20.4	4.2	6.3	75.0	90.4	89.2	4.5	5.1	4.2	0.2	0.3	0.3
**16**	22.9	4.4	8.4	75.0	92.9	89.7	1.9	2.3	1.5	0.2	0.4	0.4
**17**	26.2	2.3	9.6	70.8	93.5	88.8	2.3	3.5	0.8	0.8	0.8	0.8
**Total**	**20.1**	**5.4**	**10.7**	**70.1**	**80.7**	**79.6**	**8.4**	**11.3**	**7.2**	**1.5**	**2.6**	**2.4**

CDC—The US Center for Disease Control and Prevention, IOTF—The International Obesity Task Force reference, WHO—World Health Organization child growth standard.

A comparison of the normalized BMI value calculated using WHO formula was made between the group of girls and boys (Table 3). The mean WHO BMI z-score was significantly higher among boys than among girls (0.15 vs -0.22) and the difference was statistically significant. The mean z-score for boys from Ukraine was above zero, which means that there will be more overweight and obese people in relation to the reference WHO population.

**Table 3:** Z-score (calculated using WHO formula) statistics for boys and girls.

Sex						*p*
boys			girls			
Mean (95% c.i.)	Median	Std. dev.	Mean (95% c.i.)	Median	Std. dev.	
0.15 (0.12; 0.17)	0.19	1.32	-0.22 (-0.25; -0.20)	-0.21	1.14	0.0000***

*p* value calculated using *t* test for independence samples.

The assessment of the significance of differences in the incidence between children of both sexes was determined for each BMI category (according to WHO) (Table 4), in addition, the odds ratio for each category was determined in the group of girls relative to the group of boys. The frequency distribution of the BMI category depends on sex—overweight (19.0 vs. 11.3%) and obesity (7.0 vs. 2.6%) occur more often in the group of boys than in girls.

**Table 4:** Prevalence ratio of underweight, overweight and obesity in girls and boys aged 7–17 years (criteria of WHO).

BMI categories	Sex				
	Boys *n* (%)	PR	Girls *n* (%)	PR (95% c.i.)	*p*
Underweight	450 (5.2)	(REF)	506 (5.4)	1.04 (0.92–1.18)	0.5364
Normal	6002 (68.9)	(REF)	7610 (80.7)	1.17 (1.15–1.19)*	0.0000***
Overweight	1653 (19.0)	(REF)	1064 (11.3)	0.60 (0.55–0.64)*	0.0000***
Obesity	612 (7.0)	(REF)	246 (2.6)	0.37 (0.32–0.43)*	0.0000***

*p*–test probability value calculated using the chi-square test of independence, *p* < 0.001 (***).

PR–prevalence ratio (with 95% confidence interval), REF–reference, *p* < 0.001 (***).

Using the concept of the prevalence ratio, it was found that overweight and obesity were less common among girls (prevalence ratio 0.60 and 0.37).

## Discussion

Obesity increases the risk of many serious diseases, therefore testing and monitoring it in children is a very important element of healthcare management. Risks to children's health associated with overweight and obesity include hypertension, hyperinsulinemia, glucose intolerance, type II diabetes, dyslipidemia, an increased risk of early heart disease, and social difficulties. Overweight and obesity of children is a serious public health problem, and their accurate measurement and classification are important in determining the size of the health problem [14].

Research results indicate that the methods used to identify childhood obesity based on BMI yield different results for estimating the prevalence of obesity and / or overweight and underweight in children.

The three classifications used showed a high incidence of overweight and obesity in the study sample. IOTF and CDC classifications generated a lower incidence of overweight compared to WHO criteria, which consistently reported a higher incidence of overweight and obesity in all participants and both sexes. There were differences in the frequency of overweight and obesity between the classifications: 13% according to IOTF, 19.7% according to WHO and 14.1% according to CDC.

The IOTF classification, for which BMI was 18.5 for an adult, caused a fairly large group of children to be classified as underweight. Our results suggest that the choice of a BMI reference can significantly influence the decision to provide clinical advice or treatment, as well as estimates of the health resources required to counter underweight, overweight and obesity.

Studies on overweight and obesity in children and adolescents in southern Europe by López-Sánchez et al. showed significant differences between classifications: 32.3% according to IOTF, 37.3% according to WHO [1]. Espín Ríos et al. evaluated BMI of 178,894 students aged 2 to 14 in Spain, classifying them according to the criteria set out by WHO and IOTF. A higher incidence of overweight and obesity was found in girls compared to the present study: 42.1% according to WHO and 33.2% according to IOTF [15]. Our study has shown that the incidence of overweight according to the IOTF and CDC classification is similar (10.9% and 10.1%, respectively), while WHO indicates 15%. A similar correlation was observed by Twells and Newhook [14] examining Canadian children. They found that the CDC and IOTF classification gave similar estimates of the incidence of overweight, (19.1% and 18.2%) while WHO reported its higher incidence (26.7%).

Comparable relationships were observed in the incidence of obesity. In both our study and in that of Twells and Newhook [14], the CDC classified twice as many obese children as compared to IOTF. In our study a significantly higher incidence of childhood obesity (4.0% and 4.7%, respectively) was observed in CDC and WHO compared to IOTF (2.1%). Studies among Inuit children indicate that IOTF and CDC generated a lower incidence of obesity compared to WHO criteria, which consistently reported a higher incidence of overweight and obesity in all participants and both sexes [16]. Previous studies in various groups and countries (children and youth from France, Greece, Canada, Malaysia, Brazil, Mexico) also showed significant differences in the use of different BMI classifications, in a similar way as the current study, [17–23]. In other studies, however, the incidence of overweight and obesity was lower when the WHO reference was used, in relation to IOTF especially for teenagers living in India [3]. The incidence of overweight and obesity in the present study was higher among boys compared to girls, which is consistent with other studies [19,22].

There were differences in the frequency of underweight between the classifications: 16.8% according to IOTF, 5.3% according to WHO and 9.9% according to CDC. Similar relationships were obtained by MPhil in examining underweight among Malaysian children receiving respectively 13.1%, 4,.6%, 7,9% [11]. In Ukraine, the prevalence of underweight girls is higher than that of boys, regardless of the criteria. According to the IOTF criterion, 20.1% of girls and 13.4% of boys are underweight.

The use of BMI as a measure of childhood obesity is common, but has drawbacks because BMI does not include muscle mass, bone density, body fat distribution, bone tissue, or body water [24].

The results of this study indicate that BMI classification criteria estimate overweight and obesity in a different way, and WHO criteria result in their higher prevalence in both sexes. Our results indicate that obesity in the IOTF system was lower than in other classification systems. Identifying overweight in children is of clinical and public health significance.

International BMI classification systems are useful for comparing overweight between studies and countries, and for monitoring global trends [16].

Three international BMI classification differ in terms of the population and methods used to develop BMI reference curves for age, as well as in terms of the recommended cut-off values. Due to the large differences in the definition of obesity by different criteria, a precise definition of obesity is needed. It is recommended to assess obesity not only by BMI, but also, for example, by fat mass content [1]. Consistency in the classification of children's anthropometric status may impact clinical management and health resource planning. Suggests that international references should be carefully used to avoid a possible misclassification of the nutritional status of children.

The main strength of this study is to compare the cut-off points for BMI in a large sample of children and adolescents living in Ukraine. The research was also carried out directly; height and weight were measured and not reported in the survey. The limitation of the study is the lack of reasons for overweight and obesity and the data is limited to children and adolescents who were at school during the data collection period and cannot be extrapolated to children and adolescents who were absent on the day of the study. A limitation of this study is also the relatively low number of older adolescents (16/17 y).

## Conclusions

The study indicates that children and adolescents living in Ukraine are overweight and obese. However, estimates of prevalence of overweight and obesity differ in methods and reference cut-off points. Higher prevalence was obtained using the IOTF classification, followed by the WHO and CDC classification. In conclusion, we have noticed differences between the 3 international BMI references in the classification of underweight, overweight and obesity in children. Our results indicate the need for widespread use of at least 1 reference for comparison between populations. Depending on the system used, widely differing estimates of prevalence and relationships with age and sex are obtained. Future research should assess how well each system reflects correct body composition measurements.
